# 491. Characterizing the Association of Pre-Existing Cytomegalovirus (CMV)-specific Humoral Immunity with CMV Reactivation Risk after Hematopoietic Cell Transplantation (HCT)

**DOI:** 10.1093/ofid/ofac492.549

**Published:** 2022-12-15

**Authors:** Danniel Zamora, Elizabeth M Krantz, Laurel Joncas-Schronce, Brenda Akoto, Terry L Stevens-Ayers, Rachel A Bender Ignacio, Joshua A Hill, Mariapia Degli-Esposti, Geoffrey Hill, Michael J Boeckh

**Affiliations:** Fred Hutchinson Cancer Center, Seattle, Washington; Fred Hutch Cancer Center, Seattle, Washington; Fred Hutch Cancer Center, Seattle, Washington; Fred Hutch Cancer Center, Seattle, Washington; Fred Hutchinson Cancer Center, Seattle, Washington; University of Washington, Seattle, Washington; Fred Hutchinson Cancer Center; University of Washington, Seattle, Washington; Monash University, Melbourne, Victoria, Australia; Fred Hutchinson Cancer Center, Seattle, Washington; Fred Hutchinson Cancer Center, Seattle, Washington

## Abstract

**Background:**

There is renewed interest in CMV-specific humoral immune protection due to recent murine data suggesting that strain-specific antibodies may inhibit CMV cell-to-cell spread (*Science*. 2019; 363:288). To characterize the nature of antibody protection against CMV after HCT, we tested pre-HCT sera in a cohort of CMV D-R+ allogeneic HCT recipients from the pre-letermovir era using VirScan, a technology that measures pathogen-specific humoral immunity (IgG) at the epitope level.

**Methods:**

Patients (age 0-70 years) who received 1^st^, myeloablative, HCT for acute leukemia or myelodysplastic syndrome and who underwent PCR surveillance and preemptive therapy were included. VirScan was used on sera collected pre-HCT. Cumulative incidence curves and multivariable Cox regression analyses were used to determine the association of pre-HCT CMV antibody epitopes scores with CMV reactivation (any, >150 IU/mL, >500 IU/mL) in the first 8 weeks post-HCT.

**Results:**

170 CMV D-R+ HCT recipients were tested a median of 26 days (range 7-37 days) prior to HCT and had a median of 37 CMV antibody epitopes (IQR 29-50 epitopes, range 14-77 epitopes). The cumulative incidence of CMV reactivation was higher in patients with greater pre-HCT CMV antibody epitopes at each evaluated PCR threshold (**Figure 1**) and the effect appeared more pronounced in adults (**Figure 2**). High (≥ 4^th^ quartile vs. 1^st^ quartile), pre-HCT CMV antibody epitopes were associated with an increased risk of any CMV reactivation (adjusted Hazard Ratio [aHR]=4.1, 95% confidence interval [CI] 2.1-7.7, p< 0.001) and at >150 IU/mL (aHR=3.0, 95% CI 1.4-6.5 p=0.006) after adjusting for age, HLA-matching, and pre-HCT T-cell depletion ( >150 IU/mL only).

**Figure 1:**
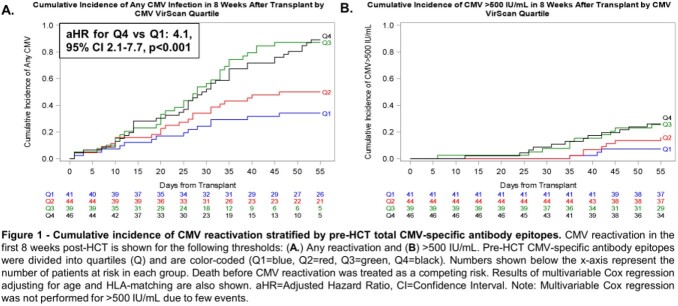

**Figure 2:**
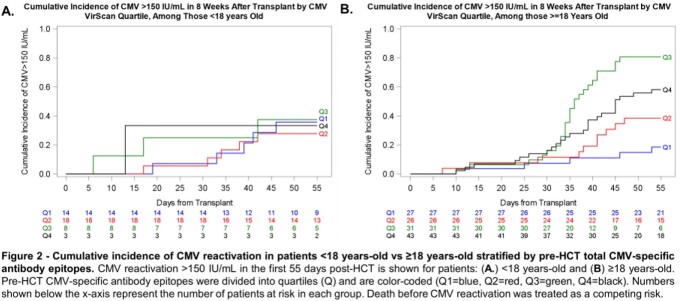

**Conclusion:**

We observed a paradoxically increased risk of CMV reactivation in patients with high pre-HCT CMV VirScan scores. It is possible that pre-HCT CMV-specific humoral immunity as measured by VirScan is reflective of the burden of latent CMV or that CMV-specific antibodies may mediate antibody-dependent enhancement after HCT and thus facilitate reactivation. Future studies are needed to validate our findings in diverse cohorts and to elucidate the underlying mechanism of increased VirScan scores and associated risks of CMV reactivation.

**Disclosures:**

**Rachel A. Bender Ignacio, MD, MPH**, Abbvie: Advisor/Consultant|SeaGen: Advisor/Consultant **Joshua A. Hill, MD**, Allovir: Advisor/Consultant|Allovir: Grant/Research Support|Covance/CSL: Advisor/Consultant|CRISPR: Advisor/Consultant|Deverra: Grant/Research Support|Gilead: Grant/Research Support|Karius: Advisor/Consultant|Karius: Grant/Research Support|Merck: Grant/Research Support|Octapharma: Advisor/Consultant|OptumHealth: Advisor/Consultant|Oxford Immunotec: Grant/Research Support|Pfizer: Advisor/Consultant|Symbio: Advisor/Consultant|Takeda: Advisor/Consultant **Geoffrey Hill, M.D., FRACP, FRCPA**, Applied Molecular Transport: Grant/Research Support|Compass Therapeutics: Grant/Research Support|Generon Corporation: Advisor/Consultant|Heat Biologics: Grant/Research Support|iTeos Therapeutics: Advisor/Consultant|iTeos Therapeutics: Grant/Research Support|Laevoroc Oncology: Grant/Research Support|NapaJen Pharma: Advisor/Consultant|Neoleukin Therapeutics: Advisor/Consultant|Serplus Technology: Grant/Research Support|Syndax Pharmaceuticals: Grant/Research Support **Michael J. Boeckh, MD PhD**, Allovir: Advisor/Consultant|Amazon: Grant/Research Support|Ansun Biopharma: Grant/Research Support|EvrysBio: Advisor/Consultant|Gates Ventures: Grant/Research Support|Gilead Sciences: Advisor/Consultant|Gilead Sciences: Grant/Research Support|GlaxoSmithKline: Advisor/Consultant|GlaxoSmithKline: Grant/Research Support|Helocyte: Advisor/Consultant|Janssen: Advisor/Consultant|Janssen: Grant/Research Support|Kyorin Pharmaceuticals: Advisor/Consultant|Merck: Advisor/Consultant|Merck: Grant/Research Support|Moderna: Advisor/Consultant|Moderna: Grant/Research Support|Regeneron: Grant/Research Support|ReViral: Advisor/Consultant|Symbio: Advisor/Consultant|Takeda: Grant/Research Support|Vir Biotechnology: Advisor/Consultant|Vir Biotechnology: Grant/Research Support.

